# Evidence for *Elizabethkingia anophelis* Transmission from Mother to Infant, Hong Kong

**DOI:** 10.3201/eid2102.140623

**Published:** 2015-02

**Authors:** Susanna K.P. Lau, Alan K.L. Wu, Jade L.L. Teng, Herman Tse, Shirly O.T. Curreem, Stephen K.W. Tsui, Yi Huang, Jonathan H.K. Chen, Rodney A. Lee, Kwok-Yung Yuen, Patrick C.Y. Woo

**Affiliations:** The University of Hong Kong, Hong Kong (S.K.P. Lau, J.L.L. Teng, H. Tse, S.O.T. Curreem, Y. Huang, J.H.K. Chen, K.-Y. Yuen, P.C.Y. Woo);; State Key Laboratory of Emerging Infectious Diseases, Research Centre of Infection and Immunology, Carol Yu Centre for Infection, Hong Kong (S.K.P. Lau, H. Tse, K.Y. Yuen, P.C.Y. Woo);; Pamela Youde Nethersole Eastern Hospital, Hong Kong (A.K.L. Wu, R.A. Lee);; School of Biomedical Sciences, The Chinese University of Hong Kong, Hong Kong (S.K.W. Tsui)

**Keywords:** Elizabethkingia anophelis, neonatal, meningitis, sepsis, vertical, transmission, maternal, bacteria, genome, mother, child, Hong Kong, sequencing

## Abstract

*Elizabethkingia anophelis*, recently discovered from mosquito gut, is an emerging bacterium associated with neonatal meningitis and nosocomial outbreaks. However, its transmission route remains unknown. We use rapid genome sequencing to investigate 3 cases of *E. anophelis* sepsis involving 2 neonates who had meningitis and 1 neonate’s mother who had chorioamnionitis. Comparative genomics revealed evidence for perinatal vertical transmission from a mother to her neonate; the 2 isolates from these patients, HKU37 and HKU38, shared essentially identical genome sequences. In contrast, the strain from another neonate (HKU36) was genetically divergent, showing only 78.6% genome sequence identity to HKU37 and HKU38, thus excluding a clonal outbreak. Comparison to genomes from mosquito strains revealed potential metabolic adaptations in *E. anophelis* under different environments. Maternal infection, not mosquitoes, is most likely the source of neonatal *E. anophelis* infections. Our findings highlight the power of genome sequencing in gaining rapid insights on transmission and pathogenesis of emerging pathogens.

## Abstract 2

Genome sequencing can provide rapid insights on transmission and pathogenesis of emerging pathogens.

Microbial genome sequencing can enhance diagnosis and control of infectious diseases (*1**,**2*). Its ultimate molecular resolution is superior to other phenotypic and genotypic tests and enables not only rapid microbial identification but also characterization of transmission events. The technique has been applied in large-scale infectious disease outbreaks such as those caused by *Escherichia coli* O104:H4, *Staphylococcus aureus*, *Streptococcus pyogenes*, *Enterococcus faecium*, *Pseudomonas aeruginosa*, *Vibrio cholerae*, and mycobacteria (*3**–**14*)*.* However, the routine application of this method in diagnostic microbiology and infection control, especially for less well-defined, emerging pathogens, is yet to be explored.

*Elizabethkingia anophelis* is a recently discovered bacterium isolated from the midgut of the *Anopheles gambiae* mosquito in 2011 (*15*)*.* The genus *Elizabethkingia* also includes *E. meningoseptica* (previously named *Chryseobacterium/Flavobacterium meningosepticum*) and *E. miricola* (*16*)*.* *E. meningoseptica* causes neonatal sepsis and infections in immunocompromised persons. *E. anophelis* has also recently been reported to cause neonatal meningitis in the Central African Republic, and a nosocomial outbreak was reported in an intensive care unit in Singapore (*17**–**19*)*.* However, the role of mosquitoes or other sources in the transmission of *E. anophelis* remains unclear.

In 2012, we encountered 3 cases of *Elizabethkingia* sepsis associated with meningitis in 2 neonates and chorioamnionitis in a neonate’s mother in a hospital in Hong Kong. Three strains of *Elizabethkingia*-like, gram-negative bacilli sharing similar phenotypic characteristics were isolated from the 3 patients, but confident identification results were not obtained by matrix-assisted laser desorption ionization/time-of-flight (MALDI-TOF) mass spectrometry and 16S rRNA gene sequencing. Moreover, clinical and microbiological data did not provide adequate clues about the possible transmission route. We therefore attempted to use draft genome sequencing to rapidly dissect transmission pathways and confirm the identity of the species. 

## Materials and Methods

### Setting and Patients

The 3 patients were hospitalized in an acute regional hospital, Pamela Youde Nethersole Eastern Hospital, which is situated in the eastern area of Hong Kong Island. This study was approved by the Institute Review Board, Hospital Authority, Hong Kong (reference HKEC-2013-051).

### Microbiological Methods

Bacterial cultures and phenotypic identification were performed according to standard protocols by using the Vitek II system (bioMérieux, Marcy l’Etoile, France). Antimicrobial drug susceptibility testing was performed by E-test method for vancomycin and Kirby-Bauer disk diffusion for other drugs; because interpretative criteria for *Elizabethkingia* were lacking, results were interpreted according to Clinical and Laboratory Standards Institute for *Pseudomonas aeruginosa* (*20*)*.* MALDI-TOF mass spectrometry was performed by the direct transfer method as described previously (*21*), with modifications by using the Bruker Daltonics microflex LT system with Reference Library Biotyper version 3.1 (Bruker Daltonik GmbH, Leipzig, Germany)*.* Full 16S rRNA gene amplification and sequencing were performed according to previously published protocols with modifications (*22**,**23*)*.* Pulsed-field gel electrophoresis (PFGE) was performed by using the CHEF Mapper XA system (Bio-Rad, Hercules, CA, USA) and restriction endonuclease *Xba*I as described previously (*8**,**22*)*.*

### Draft Genome Sequencing and Analysis

The draft genome sequences of the 3 *E. anophelis* strains were determined by high-throughput sequencing with the Illumina HiSeq 2500 system (Illumina, San Diego, CA, USA). Samples of 50 ng of genomic DNA were extracted by using a genomic DNA purification kit (QIAGEN, Hilden, Germany) from cultures grown overnight on blood agar at 37°C, as described previously (*24**,**25*)*.* Each sample was sequenced by 151-bp paired-end reads with mean library size of 350 bp. Sequencing errors were corrected by k-mer frequency spectrum analysis using SOAPec (http://soap.genomics.org.cn/about.html). De novo assembly was performed in SOAPdenovo2 (http://soap.genomics.org.cn/soapdenovo.html). Prediction of protein coding regions and automatic functional annotation was performed by using Glimmer3 (*26*) and the RAST (Rapid Annotations using Subsystem Technology) server (*27*)*.* Antibiotic resistomes were identified by using the Antibiotic Resistance Genes Database (*28*)*.* BLASTn comparisons were run in BLAST+ (http://blast.ncbi.nlm.nih.gov/Blast.cgi) with an E-value cutoff of 10.0. In addition, manual annotation was performed on putative virulence and antibiotic resistance genes by protein domain predictions and multiple sequence alignments with orthologous genes. Intergenomic distance was calculated by using Genome-to-Genome Distance Calculator 2.0 (http://ggdc.dsmz.de/distcalc2.php) (*29*)*.*

## Results

### Patients

**Figure 1:**
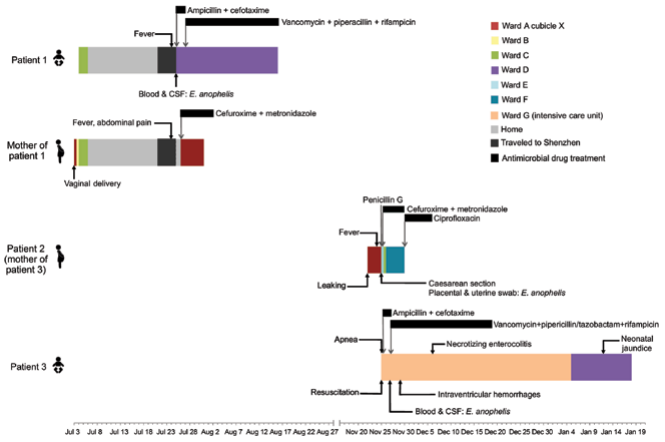
Clinical course of illness in 3 patients infected with *Elizabethkingia anophelis* in whom sepsis developed and the mother of patient 1, who had culture-negative postpartum fever, Hong Kong, 2012. Locations where patients were treated at the hospital and times when they were home are noted.CSF, cerebrospinal fluid; leaking, leaking of amniotic fluid (membrane rupture).

In July 2012, a 21-day-old male neonate (patient 1) was admitted to Pamela Youde Nethersole Eastern Hospital for fever of 1 day’s duration. He was born at the same hospital 21 days earlier at 41 weeks’ gestation by vaginal delivery and was discharged on day 3. Physical examination did not show obvious infective focus. Serum C-reactive protein (CRP) was elevated to 109 mg/L. Lumbar puncture was performed; analysis of cerebrospinal fluid (CSF) showed polymorph pleocytosis, elevated protein levels, and low glucose levels (Table). Treatment was initiated for bacterial meningitis with empirical intravenous ampicillin and cefotaxime. Blood and CSF cultures recovered a gram-negative bacillus, designated HKU36. Antimicrobial drugs were changed to vancomycin, piperacillin, and rifampin on day 3. The patient was discharged after 3 weeks of intravenous drug treatment, without neurologic sequelae (Figure 1). The neonate’s mother was admitted to the same hospital 1 day after the infant’s admission for postpartum fever, chills, rigor, and abdominal pain. Transvaginal ultrasound showed no retained gestational products. Serum CRP level was elevated to 109 mg/L; however, blood cultures were negative. She was treated with intravenous cefuroxime and metronidazole and discharged on day 6 with oral cefuroxime and metronidazole.

**Table:** Clinical characteristics and results of testing for 3 patients infected with *Elizabethkingia anophelis*, Hong Kong, 2012*

Characteristics	Patient 1	Patient 2†	Patient 3
Patient age/sex	21 d/M	33y/F	0 d/F
Signs/symptoms	Fever	Fever, PPROM	Apnea at birth
Blood test results			
Total leukocytes, × 10^9^ cells/L	16.0 (5.0–19.5)	15.2 (3.7–9.3)	5.1 (10.0–27.0)
Neutrophils, × 10^9^ cells/L	6.8 (2.0–9.5)	12.5 (1.8–6.2)	1.2 (5.0–17.0)
Lymphocytes, × 10^9^ cells/L	6.8 (2.5–11.0)	1.7 (1.0–3.2)	3.4 (3.0–10.0)
Monocytes, × 10^9^ cells/L	2.3 (0.2–1.2)	0.8 (0.2–0.7)	0 (0.5–2.0)
Hemoglobin, g/dL	14.0 (11.0–19.0)	10.7 (11.5–15.4)	16.1 (13.5–19.5)
Platelets, × 10^9^/L	180 (180–460)	241 (160–420)	186 (100–300)
C-reactive protein, mg/L	109 (<8.0)	108 (<5.0)	70.6 (<8.0)
CSF test results			
Total leukocytes, × 10^6^ cells/L	1,445	NA	5,850
Polymorphs, %	67	NA	1
Lymphocytes, %	33	NA	99
Protein, g/L	1.33 (0.15–0.45)	NA	2.69 (0.15–0.45)
Glucose, mmol/L	2.2 (2.8–4.4)	NA	1.5 (2.8–4.4)
CSF/serum glucose, %	38	NA	24
Positive culture sites for *E. anophelis*	Blood, CSF	Placental swab, uterine swab	Blood, CSF
Phenotypic characteristics of isolates			
Colony pigment	Pale yellow	None	None
Citrate utilization	Negative	Delayed positive	Delayed positive
Antimicrobial drug susceptibilities of isolates			
Ampicillin	Resistant	Resistant	Resistant
Pipercillin	Susceptible	Susceptible	Susceptible
Cefoperazone/sulbactam	Susceptible	Susceptible	Susceptible
Cefotaxime	Intermediate	Resistant	Resistant
Ceftazidime	Resistant	Resistant	Resistant
Imipenem	Resistant	Resistant	Resistant
Amikacin	Resistant	Resistant	Resistant
Gentamicin	Resistant	Resistant	Resistant
Kanamycin	Resistant	Resistant	Resistant
Streptomycin	Resistant	Resistant	Resistant
Tobramycin	Resistant	Resistant	Resistant
Ciprofloxacin	Susceptible	Susceptible	Susceptible
Moxifloxacin	Susceptible	Susceptible	Susceptible
Tetracycline	Resistant	Resistant	Resistant
Trimethoprim/sulfamethoxazole	Susceptible	Susceptible	Susceptible
Rifampin	Susceptible	Susceptible	Susceptible
Chloramphenicol	Resistant	Resistant	Resistant
Vancomycin MIC, μg/mL	16	4	4
Antimicrobial drug regimen	Ampicillin + cefotaxime; vancomycin + piperacillin + rifampin	Penicillin G; cefuroxime + metronidazole; ciprofloxacin	Ampicillin + cefotaxime; vancomycin + pipercillin/tazobactam + rifampin
Complications	None	None	Respiratory distress, intraventricular hemorrhage

*Reference ranges are shown in parentheses. PPROM, prolonged premature rupture of membranes; CSF, cerebrospinal fluid. †Mother of patient 3.

In November 2012, a 33-year-old woman in week 30 of pregnancy (patient 2) was admitted to the same hospital because of prolonged premature rupture of membranes. She stayed at the same antenatal ward and in the same cubicle as the mother of patient 1 (Figure 1). Fever developed in the patient 3 days after admission, and clinical tests showed peripheral leukocytosis with neutrophilia (Table). Serum CRP was elevated to 108 mg/L. Treatment with intravenous penicillin G was commenced, and an emergency lower segment cesarean section was performed. Placental and uterine swab cultures recovered a gram-negative bacillus, designated HKU37. Blood cultures were negative. Antimicrobial drug treatment was changed to cefuroxime and metronidazole, followed by oral ciprofloxacin for 1 week. Her fever subsided, and she was discharged on day 8.

The baby girl (patient 3) of patient 2 was pale and flaccid at birth; apnea of prematurity developed, requiring cardiopulmonary resuscitation. Peripheral leukopenia and metabolic acidosis were also detected, and serum CRP level was elevated to 70.6 mg/L. Chest radiograph showed bilateral ground-glass appearance. Lumbar puncture was performed, and CSF showed lymphocytic pleocytosis with elevated protein levels and low glucose levels (Table). Ultrasound of the brain showed grade I to II intraventricular hemorrhages. Empirical intravenous ampicillin and cefotaxime at meningitic dose was started. Blood and CSF cultures recovered a gram-negative bacillus, designated HKU38. Antimicrobial drug therapy was changed to intravenous vancomycin, piperacillin/tazobactam, and rifampin on day 3, continuing for 3 weeks. Necrotizing enterocolitis and neonatal jaundice developed, but both resolved with treatment (Figure 1). The infant was discharged on day 54 without neurologic sequelae.

### Clinical and Microbiological Investigations

The 3 isolates from these patients, HKU36–38, were nonmotile, oxidase-positive, non–glucose-fermenting, gram-negative bacilli. Their phenotypic characteristics are summarized in the Table and Technical Appendix Table 1. The isolates were identified as *E. meningoseptica* by using the Vitek II identification system (bioMérieux, Marcy L’Étoile, France). However, MALDI-TOF mass spectrometry identified strains HKU37 and HKU 38 as *E. meningoseptica* (best match to *E. meningoseptica* strain 002_NEB14 NFI, with scores of 2.106 and 2.007, respectively), whereas strain HKU36 was only identified to the genus level as *Elizabethkingia* species (best match to *E. meningoseptica* strain 002_NEB14 NFI, with score of 1.853) (Technical Appendix Figure 1). The isolates’ 16S rRNA gene sequences exhibited 99.1%–99.9% nucleotide identities to those of *E. anophelis* type strain R26^T^ (GenBank accession no. EF426425) and 97.4%–99.9% nucleotide identities to those of *E. meningoseptica* strains deposited in GenBank (GenBank accession nos. HM056770.1, GU180602.1, JQ673498.1, FJ816020, AVCQ01000012, FJ839441.1, JN201943.1, and AJ704540). 

The high sequence identities to both *E. anophelis* and *E. meningoseptica* made the species identity of the 3 strains uncertain, despite 16S rRNA gene sequencing. Moreover, the strains exhibited minor differences in phenotypes and antibiogram (Table). Further, because the mothers stayed in the same ward before delivery (although 4 months apart), concerns of a possible nosocomial outbreak were raised. However, environmental and water samples from the hospital and patients’ homes were culture-negative for *E. anophelis*. A program of enhanced infection control measures was enforced in the hospital, and no further cases were identified.

### Genome Sequencing and Comparative Analysis of *E. anophelis* Genomes

**Figure 2:**
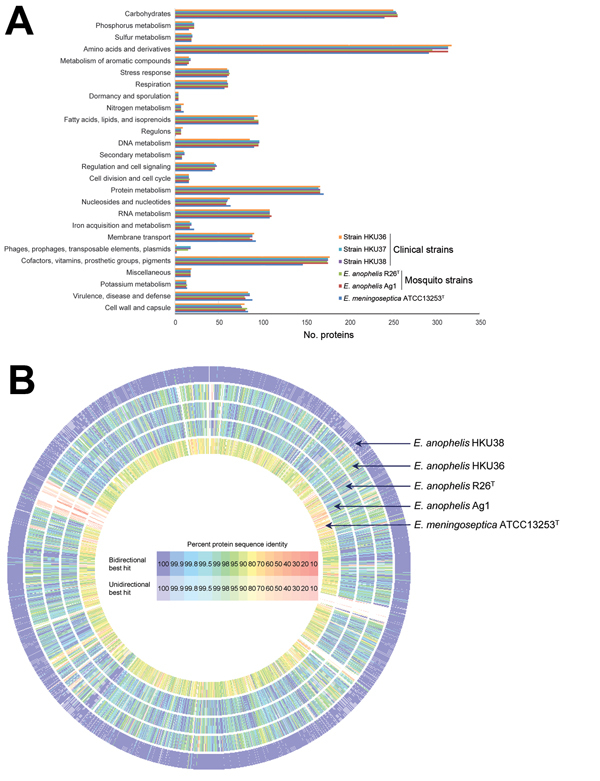
Comparison of draft genome sequence data of the 3 *Elizabethkingia anophelis* strains from patients in Hong Kong (HKU36–38), *E anophelis* type strain R26^T^, and *E. meningoseptica* type strain ATCC 13253^T^. A) Distributions of predicted coding sequence function in genomes of *E. anophelis* strains HKU36–38, *E. anophelis* type strain R26^T^, and *E. meningoseptica* type strain ATCC 13253T according to SEED Subsystems are shown. The columns indicate the number of proteins in different subsystems. B) Circular representation of sequence comparison between the draft genome of strain HKU37 and other draft genomes as labeled. Comparison generated in Rapid Annotations using Subsystem Technology (*27*). Intensity of color indicates degree of protein identity.

**Figure 3:**
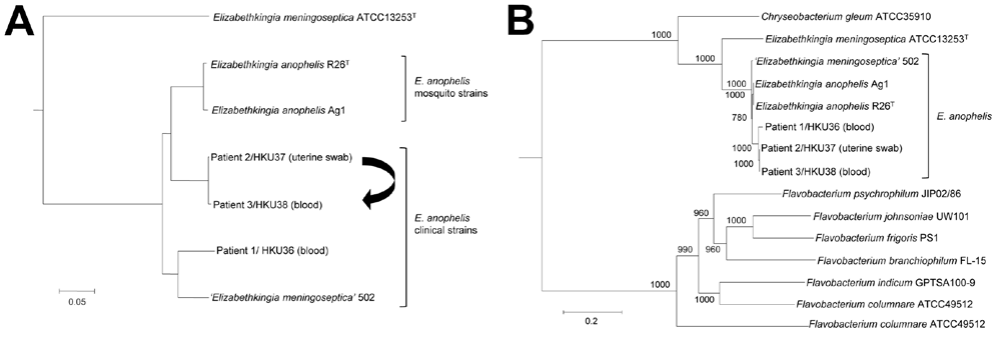
Phylogenetic trees constructed by using draft genome sequences and concatenated sequences of 69 housekeeping genes of 3 *Elizabethkingia anophelis* strains from patients in Hong Kong (HKU36–38). A) Neighbor-joining tree constructed on the basis of draft genome sequences using by using Genome-to-Genome Distance Calculator 2.0 (http://ggdc.dsmz.de/distcalc2.php; formula 1) and *Chryseobacterium gleum* ATCC 35910 as the root. Arrow indicates route of mother-to-neonate transmission. B) Maximum-likelihood tree constructed on the basis of 69 housekeeping genes, showing the relationship of *E. anophelis* strains HKU36–38 to related bacterial species, using RAxML version 7.2.8 (http://sco.h-its.org/exelixis/software.html) and *Weeksella virosa* DSM 16922 as the root. A total of 78,520 nt positions were included in the analysis. Bootstrap values were calculated from 1,000 replicates. Scale bars indicate mean number of nucleotide substitutions per site on the respective branches. Gene names and accession numbers are given as cited in GenBank (Technical Appendix Table 2). *‘E. meningoseptica’* strain 502 is a misidentified isolate that actually belongs to *E. anophelis* on the basis of draft genome sequencing.

We sequenced the draft genomes of strains HKU36–38 to investigate their genetic relatedness and confirm their species identity. Sequencing generated 11–15 million paired-end reads per strain (estimated 410–540-fold coverage). After de novo assembly, the 3 draft genomes ranged from 3.92–3.99 Mb in length (G + C content 35.4%–35.8%) and were distributed in 42–52 large (>500 bp) contigs (EMBL accession nos. CBYD010000001–CBYD010000042, CBYE010000001–CBYE010000032, CBYF010000001–CBYF010000038; Technical Appendix Table 2). These contigs contained 3,654–3,667 predicted protein-coding genes (Figure 2, panel A). Using Genome-to-Genome Distance Calculator for intergenomic distance estimation, which enabled genome-based species delineation analogous to traditional DNA–DNA hybridization method, we found that these genomes shared 78.3%–85.4% nucleotide identities to the draft genome sequence of *E. anophelis* type strain R26^T^, the initial isolate from an *Anopheles gambiae* mosquito (GenBank accession no. NZ_ANIW00000000.1). However, the genomes shared only 23.6%–23.7% nucleotide identities to the draft genome sequence of *E. meningoseptica* type strain ATCC 13253^T^ (GenBank accession no. BARD00000000.1) (Figure 2, panel B). Phylogenetic analysis using the draft genomes and concatenated sequences of 69 housekeeping genes also supported the identification of the 3 strains as *E. anophelis* (Figure 3; Technical Appendix Figure 2).

**Figure 4:**
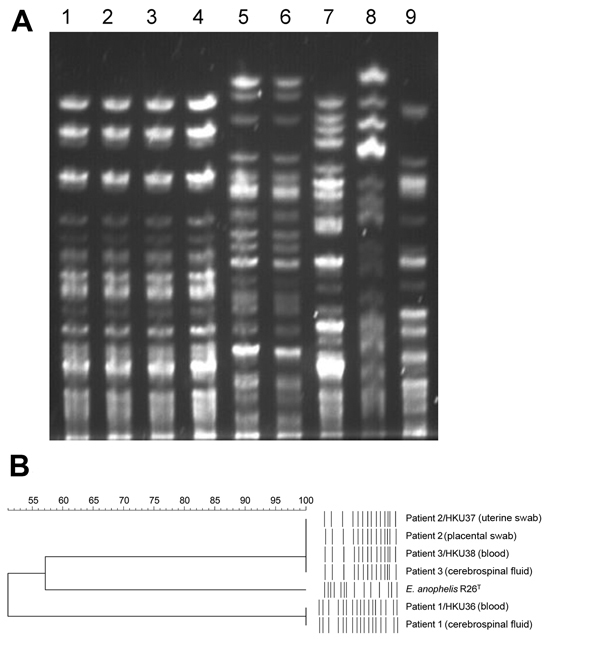
Pulsed-field gel electrophoresis (PFGE) analysis of samples from patients in Hong Kong showing 3 *Elizabethkingia anophelis* strains compared with reference *Elizabethkingia* isolates. A) PFGE performed by using CHEF Mapper XA system (Bio-Rad, Hercules, CA, USA) and restriction endonuclease *Xba*I shows that isolates from patient 2 and patient 3 are indistinguishable, wheras isolates from patient 1 possess distinct PFGE patterns. Lane 1, *E. anophelis* strain HKU37 from uterine swab specimen of patient 2; lane 2, placental swab specimen from patient 2; lane 3, *E. anophelis* strain HKU38 from blood of patient 3; lane 4, cerebrospinal fluid from patient 3; lane 5, *E. anophelis* strain HKU36 from blood of patient 1; lane 6, cerebrospinal fluid from patient 1; lane 7, *E. anophelis* type strain R26^T^; lane 8, *E. meningoseptica* type strain ATCC 13253^T^; lane 9, *E. miricola* type strain LMG22470^T^. B) Dendrogram constructed with PFGE data by similarity and clustering analysis using the Dice coefficient (1% tolerance and 0.5% optimization) and the unweighted pair-group method using average linkages with GelCompar II (Applied Maths, Sint-Martens-Latem, Belgium).

The sequences from 52 contigs of strain HKU37 demonstrated 99.4% nucleotide identity to those from 46 contigs of strain HKU38, indicating that these draft genomes are essentially identical (Figure 2, panel B, and Figure 3). The small intergenomic distance can be explained by slight differences in coverage or contig assembly; sequences of 2,000 high-coverage protein-coding genes were identical between HKU37 and HKU38. In contrast, these sequences demonstrated only 78.6% nucleotide identity to those from the 42 contigs of strain HKU36, indicating that strain HKU36 is genetically divergent (Figure 2, panel B, and Figure 3), consistent with PFGE patterns (Figure 4). Moreover, a potential genetic island consisting of conjugative transposable elements was found in strains HKU37 and HKU38 but not in HKU36. Our results exclude a clonal outbreak, but the extremely close genetic relatedness between strains HKU37 and HKU38 provides evidence for vertical transmission from patient 2 to patient 3 (mother to infant).

### Potential Virulence Factors and Resistance Genes in *E. anophelis*

The association of *E. anophelis* with neonatal meningitis in this and previous reports (*17**,**18*) suggests that the bacterium may possess virulence factors that enable it to invade the central nervous system. The 3 draft genomes we identified contain homologs of several virulence genes found in *Listeria monocytogenes*, which also causes neonatal meningitis. These genes include cell wall hydrolase A, which enables host cell invasion; phosphatidylinositol-specific phospholipase (PlcA) and listeriolysin O (LLO), which enable escape from the primary vacuole of macrophages, and genes that enable survival in the secondary vacuole of macrophages; and virulence cluster protein B (VclB). Phosphatidylinositol-specific phospholipase, listeriolysin O, and virulence cluster protein B are located in the *Listeria* pathogenicity island LIPI-1 (*30**,**31*). Moreover, the 3 genomes we identified contain homologs of arylsulfatase and genes that enable invasion of brain endothelial cells, which contribute to the ability of *Escherichia coli* to cross the blood–brain barrier in neonatal meningitis (*32*)*.*

Vertical transmission of *E. anophelis* from mother to infant also suggests that the bacterium may be able to colonize the vagina before causing ascending chorioamnionitis in the mother and neonatal infection through transplacental spread. A homologof the gene encoding agmatine deiminase, AgDI, which mediates acid tolerance in *L. monocytogenes* (*33*), was found in the *E. anophelis* genomes. Further studies may investigate the possible role of AgDI and potential adherence factors for vaginal colonization in *E. anophelis*.

Similar to *E. meningoseptica*, the 3 *E. anophelis* isolates we identified are resistant to multiple antimicrobial drugs. We found various antimicrobial resistance genes consistent with their resistance phenotypes, including metallo-β-lactamase (*bla*_GOB-1_ and *blaB14* in strain HKU36 and a novel *bla*_GOB_ and *blaB1* in strains HKU37 and HKU38) and extended-spectrum β-lactamase (*blaA*_CME-1_ in strains HKU37 and HKU38 and a potential novel *blaA*_CME-1_ variant in strain HKU36). A comparison of these β-lactamases to their corresponding orthologs in *E. meningoseptica* genomes revealed only 74%–85% amino acid identities, indicating that *E. anophelis* and related bacteria are potential reservoirs of novel β-lactamase genes (*19**,**34**,**35*)*.* Other antimicrobial resistance genes found included multidrug-resistance efflux pumps (ATP binding cassette superfamily, major facilitator superfamily, resistance-nodulation-division families, multidrug and toxic-compound extrusion family) that potentially carry resistance to a variety of compounds; chloramphenicol acetyltransferase; aminoglycoside 6-adenyltransferase; and tetracycline resistant gene. Moreover, a putative *tetX* gene was also identified; this gene encodes a predicted flavin-dependent monooxygenase with tetracycline/tigecycline-degrading activity, although the 3 strains we identified are only resistant to tetracycline but remained susceptible to other related drugs, including tigecycline.

### Comparison of Genomes from Human and Mosquito *E. anophelis* Strains

*E. anophelis* strains R26^T^ and Ag1 were isolated from mosquitoes (*35*). Compared with those strains*,* the genomes of the 3 strains we identified possessed 33 unique hypothetical proteins. Moreover, the genetic island consisting of conjugative transposable elements found in strains HKU37 and HKU38 was also absent in the mosquito strains. In contrast to the mosquito strains, which possessed genes encoding for xylose isomerase (XylA) and xylulose kinase (XylB), these 2 genes were absent in the 3 strains we identified. This finding may reflect different requirements for sugar metabolism in *E. anophelis* under different environments. Notably, despite the presence of XylA and XylB, *E. anophelis* mosquito strain R26^T^ did not produce acid from xylose (*15*). However, this finding does not exclude the strain’s ability to metabolize xylose, as D-xylulose 5-phosphate, the product of XylA and XylB, can be used as a substrate for the pentose-phosphate pathway. XylA and XylB were also absent in the genome of *E. meningoseptica* type strain ATCC 13253^T^, which suggests that mosquito strains of *E. anophelis* may be evolutionarily distinct from clinical strains of *E. anophelis* and *E. meningoseptica*. More genome sequence data from other clinical and environmental strains of *E. anophelis* may shed light on the ecology, biology, and pathogenesis of *E. anophelis*.

## Discussion

This study demonstrates the power of draft genome sequencing to rapidly dissect transmission pathways for emerging bacterial infections. Our results showed that vertical perinatal transmission had occurred from patient 2, a pregnant woman who had chorioamnionitis, to patient 3, a neonate who had early onset neonatal meningitis. The infective source for patients 2 and 3 was unlikely to have been patient 1 or his mother. However, we speculate that the mother of patient 1 might also have had *E. anophelis* chorioamnionitis, as evidenced by postpartum fever and abdominal pain, which resulted in late-onset meningitis in her son owing to fastidious bacterial growth. Although strain HKU36 did not belong to the same clone as strains HKU37/38, a polyclonal outbreak of *E. anophelis* sepsis in the labor ward, in which case an environmental source is likely, could not be excluded. 

The discovery of *E*. *anophelis* in mosquito gut has raised suspicion that mosquitoes are the source of neonatal meningitis cases in Africa (*17*). Although *Anopheles* mosquitoes are not found in Hong Kong, the role of local mosquitoes as reservoirs for *E. anophelis* remains unknown. Nonetheless, the vertical transmission demonstrated in 1 neonate makes mosquitoes unlikely as vehicles of transmission in our cases.

Our report provides genomic evidence for vertical transmission in neonatal meningitis. Whereas we cannot ascertain how the mother(s) acquired the infection, our results prompt further work to assess the importance of maternal source in neonatal meningitis caused by *E. anophelis* and other bacterial agents. Maternal colonization with Lancefield group B streptococcus (GBS) during pregnancy is the primary risk factor for early onset neonatal disease. However, direct microbiological evidence for vertical transmission is seldom available, especially for bacterial agents other than GBS. Further genomic studies may help investigate the role of vertical transmission in neonatal meningitis caused by other bacteria. Current indications for intrapartum antimicrobial drugs prophylaxis have been determined on the basis of risk factors for early onset GBS disease; therefore, intravenous penicillin G or ampicillin is often the standard empirical regimen used. However, if further research determines that the mother may also be a source of transmission for other bacterial agents, broader-spectrum antimicrobial drugs may need to be considered as treatment for intrapartum fever or prolonged rupture of membranes.

*E. anophelis* is likely an underreported bacterium because it can be easily misidentified as *E. meningoseptica*, which shares a similar phenotypic profile (*17**,**19*)*.* The *E. anophelis* isolates from the recent outbreak reported in Singapore were initially mistakenly identified as *E. meningoseptica* (*19**,**36*). Of the 3 strains we identified, 2 were misidentified as *E. meningoseptica* with MALDI-TOF mass spectrometry, the state-of-the-art technology, which is replacing conventional phenotypic identification in diagnostic laboratories. The reason for failure of MALDI-TOF mass spectrometry to identify these strains was that reference *E. anophelis* strains are lacking in existing diagnostic spectrum databases, as is the case with other less commonly encountered organisms (*21*). 

Although 16S rRNA gene sequencing should provide sufficient resolution, some strains indexed as *E. meningoseptica*, such as strains G3-1-08 and 502, were actually more closely related to *E. anophelis* than to *E. meningoseptica* in their 16S rRNA sequences (Figure 3; Technical Appendix Figure 2) (*37*)*.* These ambiguous, potentially misidentified strains may cause incorrect interpretations in suspected *E. anophelis* infections. For example, the sequence of strain HKU36 possessed 99.8% nucleotide identity to that of *E. meningingoseptica* strain G3-1-08 but only 99.1% nucleotide identity to that of *E. anophelis* strain R26^T^. Furthermore, phenotypic tests such as acid production from cellobiose and citrate utilization, previously proposed to be useful for identification of *E. anophelis* (*15*), are probably unreliable in differentiating among *Elizabethkingia* species. For example, *E. anophelis* strain R26^T^ produces acid from cellobiose, but the 3 strains we identified do not; in addition, *E. anophelis* strains R26^T^, HKU37, and HKU38, but not strain HKU36, utilize citrate (Technical Appendix Table 1). Strain HKU36 displayed higher MIC of vancomycin than did strains HKU37 and HKU38 and type strains of *E. anophelis*, *E. meningoseptica*, and *E. miricola*, which correlates with previous reports on variable vancomycin susceptibilities in *Elizabethkingia* (*38**,**39*). The species identity of the 3 strains we identified was only resolved by intergenomic comparison. Inclusion of *E. anophelis* in MALDI-TOF MS databases and rectification of 16S rRNA gene sequences of *Elizabethkingia* strains deposited in databases will enable accurate diagnosis of more *E. anophelis* infections.

The draft genome sequences we identified have enabled rapid exploration of novel β-lactamase and other antimicrobial drug resistance genes and possible virulence genes in *E. anophelis*, highlighting the potential of genome sequencing in identifying novel drug-resistance mechanisms and guiding treatment regimens for emerging, multidrug-resistant bacteria (*25**,**34**,**40*). Because previous cases of *E. anophelis* neonatal meningitis have been associated with poor outcomes (*17**,**18*), further work to elucidate the pathogenesis and antimicrobial drug resistance patterns of this emerging pathogen may help improve clinical management of illness. The findings of potential genes related to neuroinvasion and acid tolerance and the unique genetic characteristics in clinical strains of *E. anophelis* compared with mosquito strains may also provide insights on the ability of *E. anophelis* to adapt to different ecologic niches and cause neonatal infection through vertical transmission.

In conclusion, the genome data we obtained for these cases offered superior discriminatory power that supported appropriate infection control measures. The ability to distinguish different bacterial isolates often has critical implications on practical infection-control management, but different strains of the same bacterial species may not be distinguishable by their phenotypes because they reflect a tiny portion of the microbial genome. With better automation and lower costs, draft genome sequencing, which offers a short turnaround time, may replace existing typing methods such as PFGE or multilocus sequence typing for outbreak investigations.

Technical Appendix Results of matrix-assisted laser desorption ionization/time-of-flight mass spectrometry identification of 3 *Elizabethkingia anophelis* strains from patients in Hong Kong (HKU36–38), phylogenetic tree showing the relationship of HKU36–38 to closely related bacterial species using 16S rRNA gene sequence analysis, phenotypic characteristics and vancomycin susceptibilities of HKU36–38 compared with those of closely related bacterial species, results of draft genome assembly for HKU36–38, and protein names and accession numbers of the 69 housekeeping genes used for phylogenetic analysis.
