# Supplementary material for: Evidence for Elizabethkingia anophelis Transmission from Mother to Infant, Hong Kong
Source: Emerg Infect Dis. 2015 Feb;21(2):232–41. doi: 10.3201/eid2102.140623 (PMC4313635; doi:10.3201/eid2102.140623)
Supplement: Technical Appendix — Results of matrix-assisted laser desorption ionization/time-of-flight mass spectrometry identification of 3 Elizabethkingia anophelis strains from patients in Hong Kong (HKU36–38), phylogenetic tree showing the relationship of HKU36–38 to closely related bacterial species using 16S rRNA gene sequence analysis, phenotypic characteristics and vancomycin susceptibilities of HKU36–38 compared with those of closely related bacterial species, results of draft genome assembly for HKU36–38, and protein names and accession numbers of the 69 housekeeping genes used for phylogenetic analysis. [file 14-0623-Techapp-s1.pdf]

Article DOI: http://dx.doi.org/10.3201/eid2102.140623

Evidence for *Elizabethkingia anophelis* Transmission from Mother to Infant, Hong Kong

Technical Appendix

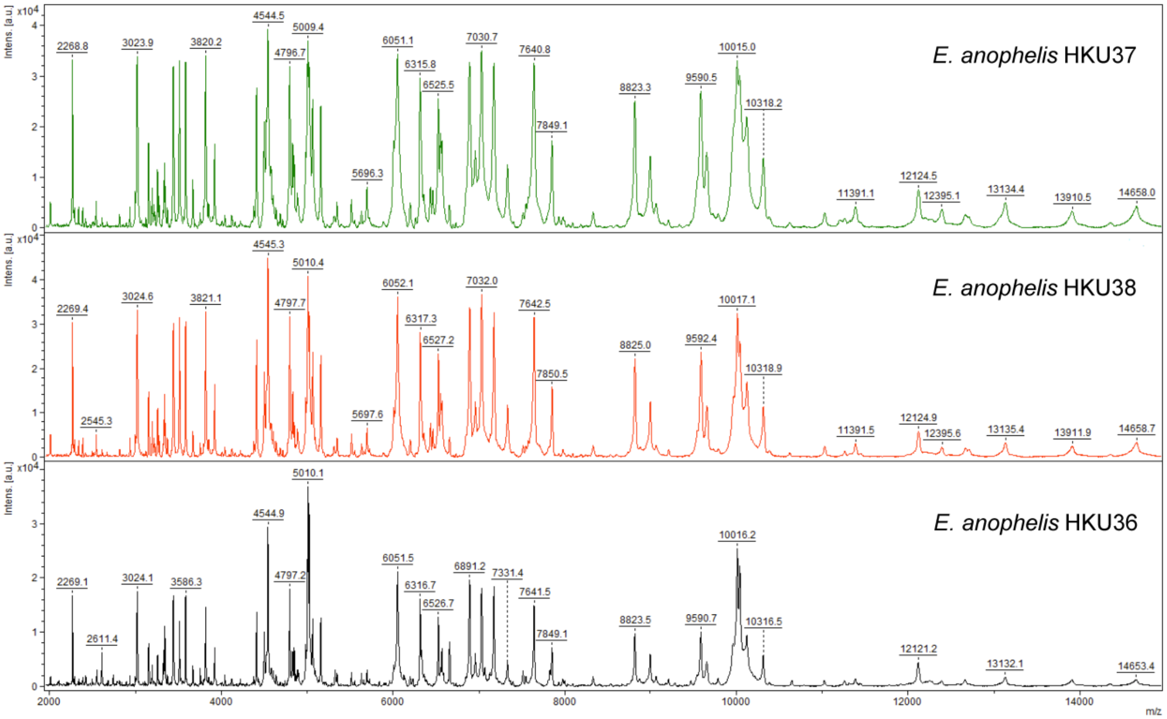

Technical Appendix Figure 1. Results of matrix-assisted laser desorption ionization/time-of-flight (MALDI-TOF) mass spectrometry identification of 3 *Elizabethkingia anophelis* strains from patients in Hong Kong (HKU36–38). In panel A, MALDI-TOF spectra of the three isolates are shown. In panel B, dendrogram was generated from hierarchical clustering of MALDI-TOF spectra of the three isolates and reference strains of *E. meningoseptica* and *E. miricola* available in database, using ClinProTools 3.0 (Bruker Daltonics, Bremen, Germany). Distances are displayed in relative units.

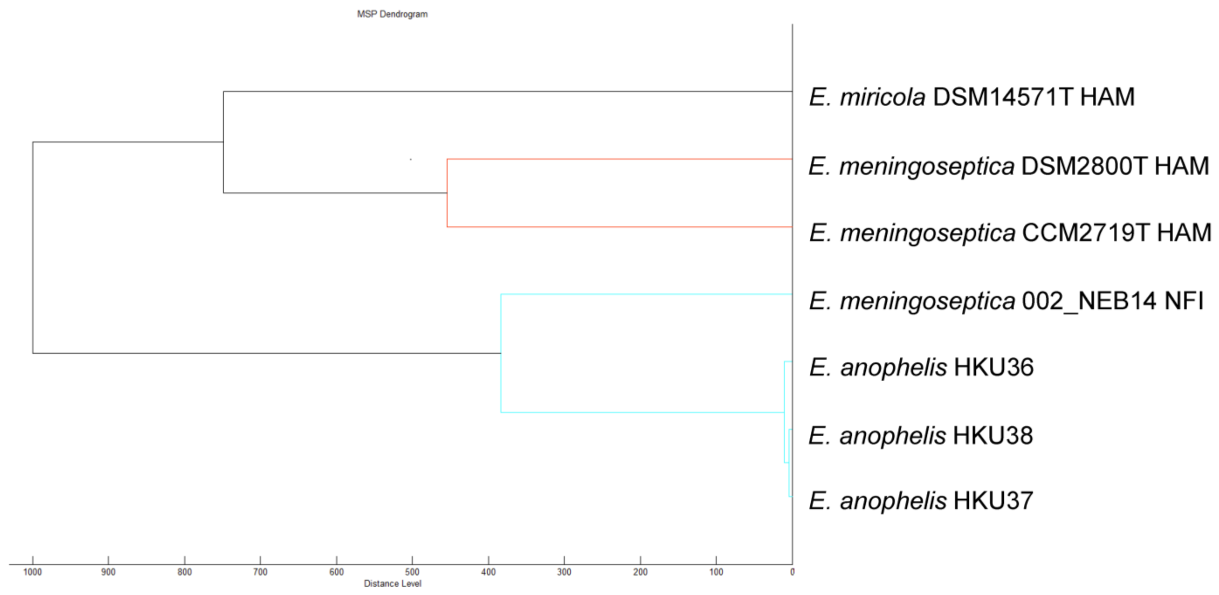

Technical Appendix Figure 2. Phylogenetic tree showing the relationship of the three *E. anophelis* strains HKU36-38 to closely related bacterial species using 16S rRNA gene sequence analysis. The tree was constructed by neighbour-joining method using *Chryseobacterium gleum* (AM232812.1) as the root. A total of 1317 nucleotide positions were included in the analysis. Bootstrap values were calculated from 1000 replicates. The scale bar indicates the estimated number of substitutions per 200 bases. Names and accession numbers are given as cited in GenBank database.

Technical Appendix Table 1. Phenotypic characteristics and vancomycin susceptibilities of the three *E. anophelis* clinical isolates compared to *E. anophelis* type strain R26^{T}, *E. meningoseptica* type strain ATCC 13253^{T} and *E. miricola* type strain LMG22470^{T}

Characteristics	*E. anophelis* strain HKU36	*E. anophelis* strain HKU37	*E. anophelis* strain HKU38	*E. anophelis* type strain R26^{T}	*E. meningoseptica* type strain ATCC 13253^{T}	*E. miricola* type strain LMG22470^{T}
Acid production from cellobiose	Negative	Negative	Negative	Positive	Negative	Weakly positive
Acid from melibiose	Negative	Negative	Negative	Negative	Positive	Weakly positive
Urea hydrolysis	Negative	Negative	Negative	Negative	Negative	Delayed positive
Citrate utilization	Negative	Delayed positive	Delayed positive	Delayed positive	Negative	Negative
Vancomycin MIC (µg/ml)	16	4	4	12	8	8

Technical Appendix Table 2. Results of draft genome assembly of the three *E. anophelis* isolates

Genome assembly data	Strain HKU36	Strain HKU37	Strain HKU38
Genome size	3.99 Mb	3.92 Mb	3.93 Mb
G + C content	35.4%	35.8%	35.8%
No. of contigs (>500 bp)	42	52	46
No. of predicted protein-coding genes	3667	3654	3662

Technical Appendix Table 3. Protein names and accession numbers of the 69 housekeeping genes used for phylogenetic analysis based on their concatenated sequences

No.	Gene	Description	*Elizabethkingia anophelis* Ag1	*Elizabethkingia anophelis* R26^{T}	*Elizabethkingia meningoseptica* ATCC 13253^{T}	'*Elizabethkingia meningoseptica*' 502	*Chryseobacterium gleum* ATCC 35910
1	*adk*	adenylate kinase	EHM99033.1	ELR78181.1	EOR29026.1	EQB93674.1	EFK36011.1
2	*aroC*	chorismate synthase protein	EHM98932.1	ELR79157.1	EOR30315.1	EQB91279.1	EFK36463.1
3	*aroE*	shikimate 5-dehydrogenase	EHM97099.1	ELR79534.1	EOR29119.1	EQB92826.1	EFK37041.1
4	*atpG*	ATP synthase gamma subunit	EHM97869.1	ELR77763.1	EOR29854.1	EQB90742.1	EFK34458.1
5	*clpX*	ATP-dependent Clp protease, ATP-binding subunit ClpX	EHM97147.1	ELR80137.1	EOR29258.1	EQB92999.1	EFK38075.1
6	*ddl*	D-alanine--D-alanine ligase	EHM98607.1	ELR80901.1	EOR30203.1	EQB91483.1	EFK37089.1
7	*dnaG*	DNA primase	EHM96290.1	ELR80281.1	EOR29602.1	EQB93431.1	EFK35645.1
8	*dnaJ*	molecular chaperone DnaJ	EHM97005.1	ELR79584.1	EOR30530.1	EQB91744.1	EFK33359.1
9	*dnaN*	DNA polymerase III beta subunit protein DnaN	EHM99096.1	ELR78117.1	EOR30852.1	EQB93614.1	EFK33728.1
10	*efp*	translation elongation factor (P) Efp	EHM96207.1	ELR79458.1	EOR31162.1	EQB92149.1	EFK33096.1
11	*engA*	GTP-binding protein EngA	EHM97976.1	ELR77871.1	EOR30120.1	EQB90633.1	EFK32926.1
12	*frr*	ribosome recycling factor	EHM97858.1	ELR77751.1	EOR28456.1	EQB90754.1	EFK34962.1
13	*ftsZ*	cell division protein	EHM96855.1	ELR79837.1	EOR29056.1	EQB92556.1	EFK37400.1
14	*gltA*	citrate synthase	EHM98067.1	ELR78920.1	EOR31281.1	EQB91049.1	EFK38000.1
15	*glyA*	serine hydroxymethyltransferase GlyA	EHM98156.1	ELR80729.1	EOR28516.1	EQB91465.1	EFK33053.1
16	*gmk*	Guanylate kinase	EHM98926.1	ELR79163.1	EOR30303.1	EQB91303.1	EFK36478.1
17	*groEL*	molecular chaperone GroEL	EHM99261.1	ELR77949.1	EOR28992.1	EQB91175.1	EFK36363.1
18	*gyrB*	DNA gyrase, subunit B	EHM97538.1	ELR80389.1	EOR28497.1	EQB90771.1	EFK35453.1
19	*hemD*	uroporphyrinogen-III synthase	EHM98513.1	ELR80039.1	EOR30745.1	EQB92637.1	EFK37767.1
20	*infC*	translation initiation factor IF-3	EHM98658.1	ELR78736.1	EOR28593.1	EQB91887.1	EFK34832.1
21	*lepA*	GTP-binding protein LepA	EHM96750.1	ELR79731.1	EOR30078.1	EQB92448.1	EFK37466.1
22	*metG*	Methionyl-tRNA synthetase	EHM97741.1	ELR80931.1	EOR30645.1	EQB92897.1	EFK35396.1
23	*murI*	glutamate racemase	EHM98602.1	ELR80896.1	EOR30198.1	EQB91488.1	EFK37084.1
24	*mutS*	DNA mismatch repair protein	EHM98490.1	ELR80062.1	EOR30770.1	EQB92613.1	EFK37559.1
25	*ndh*	NADH dehydrogenase	EHM97992.1	ELR77888.1	EOR30137.1	EQB90616.1	EFK36447.1
26	*nusA*	transcription elongation factor NusA	EHM98422.1	ELR79019.1	EOR31379.1	EQB91154.1	EFK37875.1
27	*pbpA*	penicillin-binding protein 2b	EHM96729.1	ELR79709.1	EOR31600.1	EQB92422.1	EFK37686.1
28	*pfkB*	Ribokinase	EHM99170.1	ELR78044.1	EOR30918.1	EQB93544.1	EFK35803.1
29	*pgi*	glucose-6-phosphate isomerase	EHM98320.1	ELR79898.1	EOR31480.1	EQB92300.1	EFK37251.1
30	*pgk*	phosphoglycerate kinase	EHM96766.1	ELR79747.1	EOR31635.1	EQB92464.1	EFK37486.1
31	*ppa*	manganese-dependent inorganic pyrophosphatase	EHM99474.1	ELR78442.1	EOR31031.1	EQB94005.1	EFK35025.1
32	*purE*	phosphoribosylaminoimidazole carboxylase catalytic subunit PurE	EHM96991.1	ELR79598.1	EOR30543.1	EQB91730.1	EFK33446.1
33	*purK*	phosphoribosylaminoimidazole carboxylase ATPase subunit PurK	EHM96987.1	ELR79602.1	EOR30546.1	EQB91726.1	EFK33442.1
34	*pyrG*	CTP synthetase	EHM99276.1	ELR77936.1	EOR29004.1	EQB92753.1	EFK36380.1
35	*recA*	ATP/GTP binding motif	EHM99302.1	ELR77910.1	EOR28705.1	EQB92692.1	EFK36418.1
36	*recG*	ATP-dependent DNA helicase, RecG	EHM97941.1	ELR77835.1	EOR28850.1	EQB90669.1	EFK36929.1
37	*recN*	DNA replication protein RecF	EHM98297.1	ELR79922.1	EOR31506.1	EQB92325.1	EFK37286.1
38	*ribE*	riboflavin synthase	EHM98833.1	ELR79259.1	EOR30511.1	EQB91401.1	EFK36589.1
39	*rodA*	Rod shape-determining protein rodA	EHM96730.1	ELR79710.1	EOR31601.1	EQB92423.1	EFK37687.1
40	*rplA*	50S ribosomal protein L1	EHM98921.1	ELR79169.1	EOR30297.1	EQB91309.1	EFK36283.1
41	*rplB*	50S ribosomal protein L2	EHM98093.1	ELR78947.1	EOR31308.1	EQB91075.1	EFK37967.1
42	*rplC*	50S ribosomal protein L3	EHM98096.1	ELR78950.1	EOR31311.1	EQB91078.1	EFK37964.1
43	*rplD*	50S ribosomal protein L4	EHM98095.1	ELR78949.1	EOR31310.1	EQB91077.1	EFK37965.1
44	*rplE*	50S ribosomal protein L5	EHM98085.1	ELR78938.1	EOR31299.1	EQB91066.1	EFK37976.1
45	*rplF*	50S ribosomal protein L6	EHM98082.1	ELR78935.1	EOR31296.1	EQB91063.1	EFK37979.1
46	*rplK*	50S ribosomal protein L11	EHM98920.1	ELR79170.1	EOR30296.1	EQB91310.1	EFK36282.1

No.	Gene	Description	*Elizabethkingia anophelis* Ag1	*Elizabethkingia anophelis* R26[T]	*Elizabethkingia meningoseptica* ATCC 13253[T]	'*Elizabethkingia meningoseptica*' 502	*Chryseobacterium gleum* ATCC 35910
47	*rplL*	50S ribosomal protein L7	EHM98923.1	ELR79167.1	EOR30299.1	EQB91307.1	EFK36285.1
48	*rplM*	50S ribosomal protein L13	EHM98809.1	ELR79283.1	EOR28625.1	EQB91425.1	EFK36554.1
49	*rplP*	50S ribosomal protein L16	EHM98090.1	ELR78943.1	EOR31304.1	EQB91071.1	EFK37971.1
50	*rplS*	50S ribosomal protein L19	EHM98689.1	ELR78767.1	EOR29634.1	EQB91859.1	EFK35532.1
51	*rplT*	50S ribosomal protein L20	EHM98656.1	ELR78734.1	EOR28591.1	EQB91889.1	EFK34839.1
52	*rpmA*	50S ribosomal protein L27	EHM96827.1	ELR79807.1	EOR29988.1	EQB92525.1	EFK37322.1
53	*rpoA*	DNA-directed RNA polymerase subunit alpha	EHM98071.1	ELR78924.1	EOR31285.1	EQB91053.1	EFK37989.1
54	*rpoB*	DNA-directed RNA polymerase subunit beta	EHM96382.1	ELR80997.1	EOR29540.1	EQB93109.1	EFK36289.1
55	*rpsB*	30S ribosomal protein S2	EHM98811.1	ELR79281.1	EOR28623.1	EQB91423.1	EFK36556.1
56	*rpsC*	30S ribosomal protein S3	EHM98091.1	ELR78944.1	EOR31305.1	EQB91072.1	EFK37970.1
57	*rpsE*	30S ribosomal protein S5	EHM98080.1	ELR78933.1	EOR31294.1	EQB91061.1	EFK37981.1
58	*rpsI*	30S ribosomal protein S9	EHM98810.1	ELR79282.1	EOR28624.1	EQB91424.1	EFK36555.1
59	*rpsJ*	30S ribosomal protein S10	EHM98112.1	ELR78966.1	EOR31325.1	EQB91094.1	EFK37949.1
60	*rpsK*	30S ribosomal protein S11	EHM98073.1	ELR78926.1	EOR31287.1	EQB91055.1	EFK37987.1
61	*rpsM*	30S ribosomal protein S13	EHM98074.1	ELR78927.1	EOR31288.1	EQB91056.1	EFK37986.1
62	*rpsS*	30S ribosomal protein S19	EHM98092.1	ELR78946.1	EOR31307.1	EQB91074.1	EFK37968.1
63	*sucA*	2-oxoglutarate dehydrogenase decarboxylase component	EHM99425.1	ELR78493.1	EOR30239.1	EQB93956.1	EFK34424.1
64	*alaS*	Alanyl-RNA synthetase	EHM96283.1	ELR80290.1	EOR29610.1	EQB93424.1	EFK35625.1
65	*tktA*	transketolase	EHM96930.1	ELR79660.1	EOR30604.1	EQB91663.1	EFK36754.1
66	*tpiA*	triosephosphate isomerase	EHM96304.1	ELR80267.1	EOR29585.1	EQB93446.1	EFK35642.1
67	*trpC*	indole-3-glycerol-phosphate synthase	EHM99573.1	ELR78343.1	EOR30932.1	EQB94109.1	EFK35468.1
68	*tsf*	elongation factor Ts	EHM98813.1	ELR79279.1	EOR28621.1	EQB91421.1	EFK36564.1
69	*uvrA*	excinuclease ABC A subunit UvrA	EHM97607.1	ELR80434.1	EOR29973.1	EQB90839.1	EFK34489.1

No.	Gene	Description	*Flavobacterium branchiophilum* FL-15	*Flavobacterium columnare* ATCC 49512	*Flavobacterium frigoris* PS1	*Flavobacterium indicum* GPTSA100-9	*Flavobacterium johnsoniae* UW101	*Flavobacterium rivuli* DSM21788	*Flavobacterium psychrophilum* JIP02/86	*Weeksella virosa* DSM16922
1	*adk*	adenylate kinase	YP_004843328.1	YP_004942855.1	EIA10324.1	YP_005356587.1	YP_001192775.1	WP_020212144.1	YP_001296253.1	YP_004238641.1
2	*aroC*	chorismate synthase protein	YP_004843241.1	YP_004941761.1	EIA07987.1	YP_005357433.1	YP_001196894.1	WP_020215022.1	YP_001295684.1	YP_004239059.1
3	*aroE*	shikimate 5-dehydrogenase	YP_004844535.1	YP_004940842.1	EIA08302.1	YP_005357053.1	YP_001194935.1	WP_020211950.1	YP_001296643.1	YP_004237502.1
4	*atpG*	ATP synthase gamma subunit	YP_004842530.1	YP_004942235.1	EIA10015.1	YP_005358512.1	YP_001193412.1	WP_020214871.1	YP_001297311.1	YP_004238492.1
5	*clpX*	ATP-dependent Clp protease, ATP-binding subunit ClpX	YP_004843243.1	YP_004941516.1	EIA09493.1	YP_005356442.1	YP_001194054.1	WP_020213425.1	YP_001296948.1	YP_004238734.1
6	*ddl*	D-alanine--D-alanine ligase	YP_004844026.1	YP_004941995.1	EIA08323.1	YP_005357292.1	YP_001194964.1	WP_020211540.1	YP_001295574.1	YP_004237806.1
7	*dnaG*	DNA primase	YP_004844608.1	YP_004941419.1	EIA09098.1	YP_005358326.1	YP_001194141.1	WP_020213753.1	YP_001296936.1	YP_004239226.1
8	*dnaJ*	molecular chaperone DnaJ	YP_004844765.1	YP_004942990.1	EIA07801.1	YP_005356894.1	YP_001194720.1	WP_020211830.1	YP_001295590.1	YP_004239321.1
9	*dnaN*	DNA polymerase III beta subunit protein DnaN	YP_004843556.1	YP_004942175.1	EIA07252.1	YP_005357766.1	YP_001195061.1	WP_020211708.1	YP_001295990.1	YP_004237708.1
10	*efp*	translation elongation factor (P) Efp	YP_004844349.1	YP_004940702.1	EIA07534.1	YP_005357424.1	YP_001195241.1	WP_020211751.1	YP_001295882.1	YP_004237795.1
11	*engA*	GTP-binding protein EngA	YP_004844816.1	YP_004942744.1	EIA09193.1	YP_005358203.1	YP_001194230.1	WP_020213093.1	YP_001296829.1	YP_004238212.1
12	*frr*	ribosome recycling factor	YP_004845164.1	YP_004941270.1	EIA09266.1	YP_005358624.1	YP_001192929.1	WP_020213359.1	YP_001295365.1	YP_004239294.1
13	*ftsZ*	cell division protein	YP_004843062.1	YP_004941396.1	EIA09118.1	YP_005358278.1	YP_001194165.1	WP_020213744.1	YP_001296917.1	YP_004237973.1
14	*gltA*	citrate synthase	YP_004844776.1	YP_004941349.1	EIA10264.1	YP_005356640.1	YP_001192722.1	WP_020213492.1	YP_001296202.1	YP_004238792.1
15	*glyA*	serine hydroxymethyltransferase GlyA	YP_004844321.1	YP_004941760.1	EIA08001.1	YP_005357155.1	YP_001195743.1	WP_020211164.1	YP_001295601.1	YP_004238585.1
16	*gmk*	Guanylate kinase	YP_004845194.1	YP_004943171.1	EIA10106.1	YP_005357890.1	YP_001193096.1	WP_020212935.1	YP_001295279.1	YP_004237674.1
17	*groEL*	molecular chaperone GroEL	YP_004844224.1	YP_004942930.1	EIA09161.1	YP_005358225.1	YP_001194193.1	WP_020214765.1	YP_001296851.1	YP_004237859.1

No.	Gene	Description	*Flavobacterium branchiophilum* FL-15	*Flavobacterium columnare* ATCC 49512	*Flavobacterium frigoris* PS1	*Flavobacterium indicum* GPTSA100-9	*Flavobacterium johnsoniae* UW101	*Flavobacterium rivuli* DSM21788	*Flavobacterium psychrophilum* JIP02/86	*Weeksella virosa* DSM16922
18	*gyrB*	DNA gyrase, subunit B	YP_004843747.1	YP_004941981.1	EIA08992.1	YP_005357836.1	YP_001194601.1	WP_020212750.1	YP_001295451.1	YP_004238535.1
19	*hemD*	uroporphyrinogen-III synthase	YP_004843024.1	YP_004943282.1	EIA09279.1	YP_005358593.1	YP_001192910.1	WP_020213377.1	YP_001295118.1	YP_004238559.1
20	*infC*	translation initiation factor IF-3	YP_004844738.1	YP_004940781.1	EIA10498.1	YP_005356931.1	YP_001192382.1	WP_020215189.1	YP_001295810.1	YP_004238548.1
21	*lepA*	GTP-binding protein LepA	YP_004844927.1	YP_004942415.1	EIA10160.1	YP_005358664.1	YP_001193139.1	WP_020212956.1	YP_001297099.1	YP_004238325.1
22	*metG*	Methionyl-tRNA synthetase	YP_004845262.1	YP_004942277.1	EIA09859.1	YP_005358540.1	YP_001193210.1	WP_020214992.1	YP_001294995.1	YP_004238214.1
23	*murI*	glutamate racemase	YP_004844480.1	YP_004941529.1	EIA09443.1	YP_005356427.1	YP_001194038.1	WP_020213207.1	YP_001296963.1	YP_004239318.1
24	*mutS*	DNA mismatch repair protein	YP_004842808.1	YP_004941508.1	EIA09506.1	YP_005358374.1	YP_001194074.1	WP_020212380.1	YP_001295324.1	YP_004238747.1
25	*ndh*	NADH dehydrogenase	YP_004844830.1	YP_004941835.1	EIA07632.1	YP_005356809.1	YP_001195192.1	WP_020211712.1	YP_001296799.1	YP_004239336.1
26	*nusA*	transcription elongation factor NusA	YP_004842699.1	YP_004942659.1	EIA09378.1	YP_005356369.1	YP_001193980.1	WP_020213406.1	YP_001295398.1	YP_004238583.1
27	*pbpA*	penicillin-binding protein 2b	YP_004842581.1	YP_004941462.1	EIA09649.1	YP_005356495.1	YP_001193806.1	WP_020212211.1	YP_001297275.1	YP_004238739.1
28	*pfkB*	Ribokinase	YP_004845172.1	YP_004943205.1	EIA10148.1	YP_005358652.1	YP_001193483.1	WP_020213838.1	YP_001295133.1	YP_004238344.1
29	*pgi*	glucose-6-phosphate isomerase	YP_004842585.1	YP_004942598.1	EIA10132.1	YP_005358681.1	YP_001193089.1	WP_020213880.1	YP_001295083.1	YP_004238140.1
30	*pgk*	phosphoglycerate kinase	YP_004842563.1	YP_006194203.1	EIA09930.1	YP_005356095.1	YP_001193329.1	WP_020214854.1	YP_001294958.1	YP_004238305.1
31	*ppa*	manganese-dependent inorganic pyrophosphatase	YP_004842932.1	YP_004941449.1	EIA09673.1	YP_005356512.1	YP_001193829.1	WP_020212182.1	YP_001295270.1	YP_004238254.1
32	*purE*	phosphoribosylaminoimidazole carboxylase catalytic subunit PurE	YP_004844610.1	YP_004942860.1	EIA10328.1	YP_005356582.1	YP_001192781.1	WP_020213481.1	YP_001296257.1	YP_004238035.1
33	*purK*	phosphoribosylaminoimidazole carboxylase ATPase subunit PurK	YP_004844366.1	YP_004942857.1	EIA10327.1	YP_005356584.1	YP_001192780.1	WP_020213480.1	YP_001296256.1	YP_004238034.1
34	*pyrG*	CTP synthetase	YP_004843652.1	YP_004941223.1	EIA09061.1	YP_005357320.1	YP_001194729.1	WP_020212691.1	YP_001296767.1	YP_004237877.1
35	*recA*	ATP/GTP binding motif	YP_004844934.1	YP_004942626.1	EIA09935.1	YP_005358697.1	YP_001193340.1	WP_020212902.1	YP_001297102.1	YP_004238617.1
36	*recG*	ATP-dependent DNA helicase, RecG	YP_004844110.1	YP_004941087.1	EIA07485.1	YP_005357092.1	YP_001195275.1	WP_020211315.1	YP_001295915.1	YP_004239004.1
37	*recN*	DNA replication protein RecF	YP_004844143.1	YP_004942028.1	EIA08410.1	YP_005357867.1	YP_001195798.1	WP_020211306.1	YP_001296710.1	YP_004239260.1
38	*ribE*	riboflavin synthase	YP_004842654.1	YP_004942234.1	EIA09229.1	YP_005358530.1	YP_001193004.1	WP_020215095.1	YP_001297239.1	YP_004238363.1
39	*rodA*	Rod shape-determining protein rodA	YP_004842582.1	YP_004941463.1	EIA09648.1	YP_005356494.1	YP_001193805.1	WP_020212212.1	YP_001297276.1	YP_004238738.1
40	*rplA*	50S ribosomal protein L1	YP_004843132.1	YP_004942684.1	EIA08871.1	YP_005358134.1	YP_001194291.1	WP_020211976.1	YP_001296077.1	YP_004238016.1
41	*rplB*	50S ribosomal protein L2	YP_004844803.1	YP_004942809.1	EIA10291.1	YP_005356613.1	YP_001192749.1	WP_020213637.1	YP_001296230.1	YP_004238655.1
42	*rplC*	50S ribosomal protein L3	YP_004844806.1	YP_004942812.1	EIA10294.1	YP_005356610.1	YP_001192752.1	WP_020213634.1	YP_001296233.1	YP_004238652.1
43	*rplD*	50S ribosomal protein L4	YP_004844805.1	YP_004942811.1	EIA10293.1	YP_005356611.1	YP_001192751.1	WP_020213635.1	YP_001296232.1	YP_004238653.1
44	*rplE*	50S ribosomal protein L5	YP_004844794.1	YP_004942800.1	EIA10282.1	YP_005356622.1	YP_001192740.1	WP_020213645.1	YP_001296221.1	YP_004238664.1
45	*rplF*	50S ribosomal protein L6	YP_004844791.1	YP_004942797.1	EIA10279.1	YP_005356625.1	YP_001192737.1	WP_020213648.1	YP_001296218.1	YP_004238672.1
46	*rplK*	50S ribosomal protein L11	YP_004843131.1	YP_004942685.1	EIA08870.1	YP_005358135.1	YP_001194290.1	WP_020211977.1	YP_001296078.1	YP_004238017.1
47	*rplL*	50S ribosomal protein L7	YP_004843134.1	YP_004942682.1	EIA08873.1	YP_005358132.1	YP_001194293.1	WP_020211974.1	YP_001296075.1	YP_004238014.1
48	*rplM*	50S ribosomal protein L13	YP_004842586.1	YP_004941282.1	EIA09368.1	YP_005356488.1	YP_001193963.1	WP_020214767.1	YP_001295385.1	YP_004238816.1
49	*rplP*	50S ribosomal protein L16	YP_004844799.1	YP_004942805.1	EIA10287.1	YP_005356617.1	YP_001192745.1	WP_020213641.1	YP_001296226.1	YP_004238659.1
50	*rplS*	50S ribosomal protein L19	YP_004845156.1	YP_004943082.1	EIA08132.1	YP_005356916.1	YP_001194529.1	WP_020212560.1	YP_001296742.1	YP_004237673.1
51	*rplT*	50S ribosomal protein L20	YP_004844740.1	YP_004940779.1	EIA10500.1	YP_005356929.1	YP_001192384.1	WP_020215187.1	YP_001295808.1	YP_004238550.1
52	*rpmA*	50S ribosomal protein L27	YP_004842988.1	YP_004940654.1	EIA09345.1	YP_005356480.1	YP_001193897.1	WP_020212342.1	YP_001297073.1	YP_004238403.1
53	*rpoA*	DNA-directed RNA polymerase subunit alpha	YP_004844780.1	YP_004942786.1	EIA10269.1	YP_005356636.1	YP_001192726.1	WP_020213657.1	YP_001296207.1	YP_004238683.1
54	*rpoB*	DNA-directed RNA polymerase subunit beta	YP_004843135.1	YP_004942681.1	EIA08874.1	YP_005358131.1	YP_001194294.1	WP_020211973.1	YP_001296074.1	YP_004238013.1
55	*rpsB*	30S ribosomal protein S2	YP_004842588.1	YP_004941280.1	EIA09366.1	YP_005356486.1	YP_001193961.1	WP_020214769.1	YP_001295383.1	YP_004238818.1
56	*rpsC*	30S ribosomal protein S3	YP_004844800.1	YP_004942806.1	EIA10288.1	YP_005356616.1	YP_001192746.1	WP_020213640.1	YP_001296227.1	YP_004238658.1
57	*rpsE*	30S ribosomal protein S5	YP_004844789.1	YP_004942795.1	EIA10277.1	YP_005356627.1	YP_001192735.1	WP_020213650.1	YP_001296216.1	YP_004238674.1
58	*rpsI*	30S ribosomal protein S9	YP_004842587.1	YP_004941281.1	EIA09367.1	YP_005356487.1	YP_001193962.1	WP_020214768.1	YP_001295384.1	YP_004238817.1
59	*rpsJ*	30S ribosomal protein S10	YP_004844837.1	YP_004942813.1	EIA10295.1	YP_005356609.1	YP_001192753.1	WP_020213633.1	YP_001296234.1	YP_004238651.1
60	*rpsK*	30S ribosomal protein S11	YP_004844782.1	YP_004942788.1	EIA10271.1	YP_005356634.1	YP_001192728.1	WP_020213655.1	YP_001296209.1	YP_004238681.1
61	*rpsM*	30S ribosomal protein S13	YP_004844783.1	YP_004942789.1	EIA10272.1	YP_005356633.1	YP_001192729.1	WP_020213654.1	YP_001296210.1	YP_004238680.1

No.	Gene	Description	*Flavobacterium branchiophilum* FL-15	*Flavobacterium columnare* ATCC 49512	*Flavobacterium frigoris* PS1	*Flavobacterium indicum* GPTSA100-9	*Flavobacterium johnsoniae* UW101	*Flavobacterium rivuli* DSM21788	*Flavobacterium psychrophilum* JIP02/86	*Weeksella virosa* DSM16922
62	*rpsS*	30S ribosomal protein S19	YP_004844802.1	YP_004942808.1	EIA10290.1	YP_005356614.1	YP_001192748.1	WP_020213638.1	YP_001296229.1	YP_004238656.1
63	*sucA*	2-oxoglutarate dehydrogenase decarboxylase component	YP_004842676.1	YP_004942519.1	EIA09805.1	YP_005356282.1	YP_001193607.1	WP_020212406.1	YP_001297263.1	YP_004237778.1
64	*alaS*	Alanyl-RNA synthetase	YP_004844354.1	YP_004942759.1	EIA09202.1	YP_005358194.1	YP_001194245.1	WP_020213083.1	YP_001296809.1	YP_004238610.1
65	*tktA*	transketolase	YP_004844684.1	YP_004940771.1	EIA10540.1	YP_005357074.1	YP_001192406.1	WP_020211810.1	YP_001295603.1	YP_004237843.1
66	*tpiA*	triosephosphate isomerase	YP_004843541.1	YP_004942119.1	EIA08379.1	YP_005356847.1	YP_001195837.1	WP_020212471.1	YP_001296314.1	YP_004238777.1
67	*trpC*	indole-3-glycerol-phosphate synthase	YP_004844164.1	YP_004940849.1	EIA08447.1	YP_005357469.1	YP_001197212.1	WP_020212759.1	YP_001295434.1	YP_004238220.1
68	*tsf*	elongation factor Ts	YP_004842589.1	YP_004941279.1	EIA09365.1	YP_005356485.1	YP_001193960.1	WP_020214770.1	YP_001295382.1	YP_004238819.1
69	*uvrA*	excinuclease ABC A subunit UvrA	YP_004843715.1	YP_004940827.1	EIA07181.1	YP_005357832.1	YP_001196827.1	WP_020211841.1	YP_001295765.1	YP_004237701.1
